# Comparing simulations of actin filament compression reveals tradeoff between computational cost and capturing supertwist

**DOI:** 10.17912/micropub.biology.001347

**Published:** 2025-01-21

**Authors:** Blair Lyons, Saurabh S. Mogre, Karthik Vegesna, Jessica S. Yu, Mark Hansen, Aadarsh Raghunathan, Graham T. Johnson, Eran Agmon, Matthew Akamatsu

**Affiliations:** 1 Allen Institute for Cell Science, Seattle, WA, USA; 2 Department of Biology, University of Washington, Seattle, WA, USA; 3 Center for Cell Analysis and Modeling, University of Connecticut School of Medicine, Farmington, CT, USA

## Abstract

The dynamic bending and twisting of actin drives numerous cellular processes. To compare how different spatial scales in actin models capture these dynamics, we developed two models of actin filaments: one at monomer-scale using ReaDDy and one at fiber-scale using Cytosim. Simulating filament compression across a range of velocities, we found a divergence between the monomer- and fiber-scale simulations; notably, the monomer-scale simulations more effectively captured filament supertwist, characteristic of helical structure, but at a higher computational cost. Such comparisons can aid in designing more efficient and accurate multi-scale biological models. Interactive visualizations at https://simularium.github.io/subcell-website.

**Figure 1. Monomer-resolution simulations of compressing actin filaments capture out-of-plane filament twist more than fiber-scale simulations:**
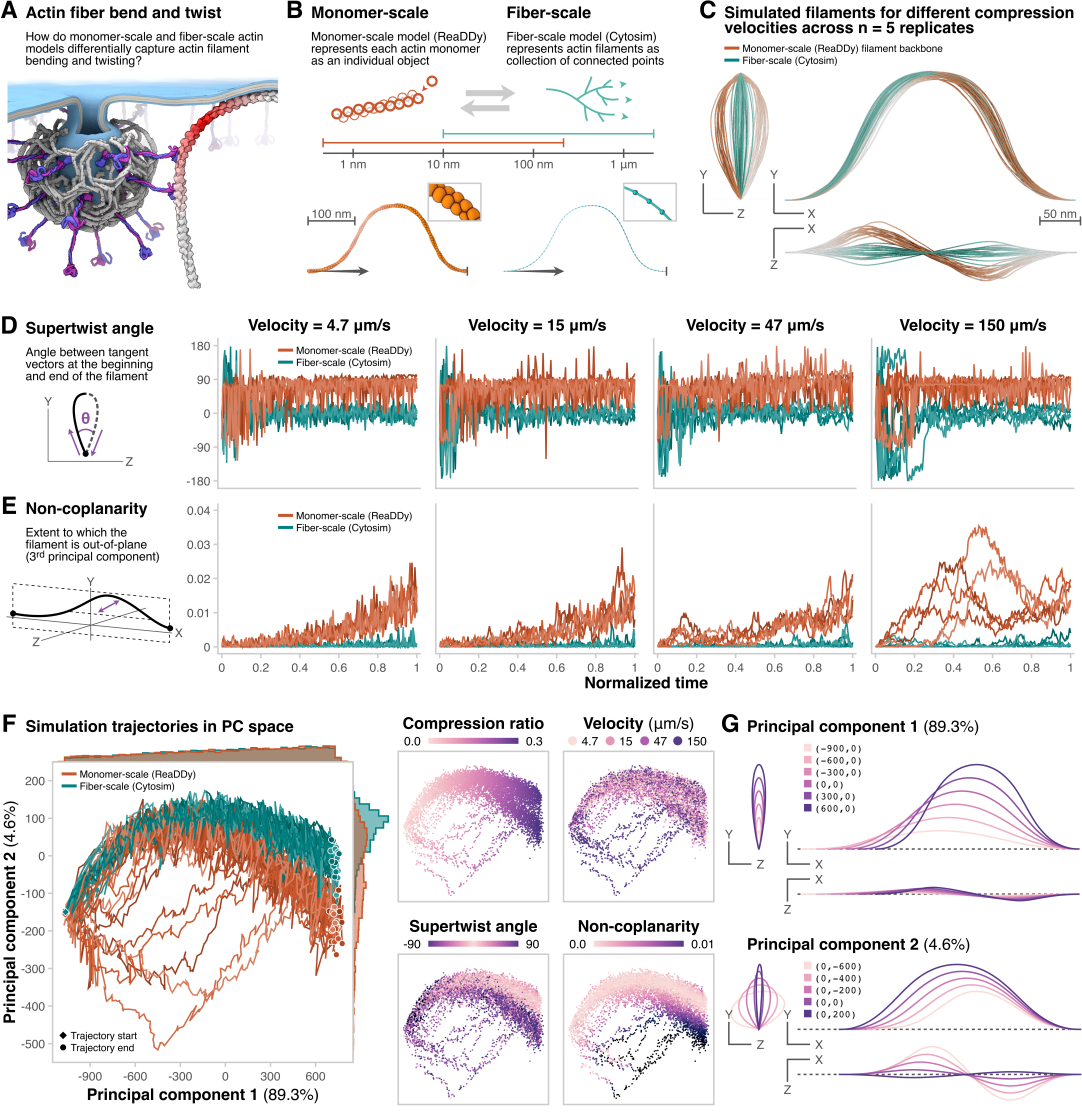
( **A** ) Diagram illustrating the role of actin filament bending and twisting in endocytosis. ( **B** ) ( *Top* ) Diagram of actin filaments modeled at monomer-scale and fiber-scale, with corresponding physical length scales. ( *Bottom* ) Snapshots of actin filament simulations compressed to 70% of initial end-to-end distance visualized in Simularium. ( **C** ) Traces of filament shape compressed to 70% of initial end-to-end distance. ReaDDy filament shape calculated from monomer positions. The top left, top right, and bottom right views show projection in the Y-Z, Y-X, and Z-X plane, respectively. Depth is rendered using atmospheric perspective, where lighter colors indicate the filament is farther from the viewer. Plots for each simulator, velocity, and replicate describing ( **D** ) Peak asymmetry and ( **E** ) Non-coplanarity over normalized compression time, and ( **F** ) Trajectories of filaments in principal component (PC) space. The start or end of each trajectory is marked by a diamond or circle, respectively. Histograms show PC1 and PC2 distributions for each simulator. ( *Right* ) Scatter plots of filaments projected into PC space, colored by features. ( **G** ) Latent walks for filament shape along PC1 ( *top* ) and PC2 ( *bottom* ).

## Description

Fundamental cellular processes such as endocytosis, cell motility, and cytokinesis rely on a cell's ability to produce force. The actin cytoskeleton plays a central role in force production in these processes ( Figure 1A ). There have been many independent efforts to model these processes using various spatial simulation methods that emphasize different spatiotemporal scales (Akamatsu et al., 2020; De La Cruz et al., 2010; Garner & Theriot, 2022; Holz et al., 2022; McDargh et al., 2023; Ni & Papoian, 2021) . However, each simulation method has limitations due to its particular implementation and assumptions characteristic of the simulated length scale. Quantitatively comparing outcomes of actin filament models that use different methods at different scales is a necessary step toward understanding the strengths and limitations of methods, reproducing results, and eventually, enabling multi-scale modeling.

*Cytosim* (Nedelec & Foethke, 2007) is a simulation engine that specializes in cytoskeleton simulations, representing actin filaments as a chain of connected Brownian particles. While effective for modeling large systems of flexible filaments, Cytosim lacks the ability to capture filament helicity. On the other hand, *ReaDDy* (Schöneberg & Noé, 2013) , a generalized particle-based reaction-diffusion simulator, can be used to model actin filaments as coarse-grained monomers, providing greater detail and capturing the helical nature of actin filaments, albeit at a higher computational cost ( Figure 1B ).

Here, we compared the shape of actin filaments modeled by these two different simulation engines. We sought to determine regimes in which each simulator captured the 3D mechanics of compressing actin filaments. To do this, we simulated compression of a 500 nm actin filament at four compression velocities, spanning a range of velocities that were physiologically and computationally feasible (Bibeau et al., 2023; Chakrabarti et al., 2020; Roffay et al., 2021; Wioland et al., 2019) .

These simulations showed systematic differences in the shapes of these filaments over time. The monomeric filaments simulated using ReaDDy had a consistent directional out-of-plane behavior, indicative of filament supertwist only captured with monomer-scale resolution ( Figure 1C ). The Cytosim simulations showed very little out-of-plane behavior at any compression velocity, and when they did, it was just as likely to be in either direction ( Figure 1C ).

We next calculated metrics to capture differences in these filaments' shapes. **Supertwist angle** is the signed angle between the projections of the initial and final tangent vectors along the compression axis ( Figure 1D ). This metric is conceptually related to writhe (Berger & Prior, 2006) . The supertwist angle sign was determined by the chirality of the filament coil, with a right-handed coil denoting positive chirality. The supertwist angle showed transient fluctuations during initial compression for both simulators, but stabilized around a characteristic simulator-dependent value at later time points. We found that the supertwist angle for filaments simulated by ReaDDy was consistently positive, compared to the supertwist angle for filaments simulated by Cytosim, which fluctuated around zero ( Figure 1E ). This observation corresponds to the twist-bend coupling observed in theory and simulations by De La Cruz et al. (De La Cruz et al., 2010) . **Non-coplanarity** is the variance explained by the third principal component (PC) calculated on individual filament coordinates at a single time point. The first two PCs capture the dominant in-plane variability of the filament shape. We found that during compression, the non-coplanarity of filaments simulated by ReaDDy was systematically higher than filaments simulated by Cytosim ( Figure 1E ).

We then conducted Principal Component Analysis on the set of aligned filament shapes across all time points to better understand the divergence in filament morphology. Values of the first PC (PC1) remained similar between the simulators, while the second PC (PC2) values initially overlapped and then diverged over time ( Figure 1F ). PC1 correlated with compression ratio, which captures the distance between end points of the actin filament, and is linearly correlated with simulation time ( Figure 1F ). PC2 largely correlated with supertwist angle and non-coplanarity, with ReaDDY showing higher supertwist and non-coplanarity compared to Cytosim ( Figure 1F ). For the filaments simulated by ReaDDy, the fastest velocity of compression corresponded to the highest values of PC2 ( Figure 1F ).

Inverse transforms of the PCs into filament shapes showed that PC1 captured filament compression, while PC2 captured filament non-coplanarity and supertwist angle ( Figure 1G ). Inverse transforms also revealed that the highest values of PC2 correspond to supertwist in the opposite direction. Only Cytosim simulations show this behavior, likely because directional helicity is not enforced in Cytosim. Overall, we conclude that simulating filament compression at monomer scale captures directional out-of-plane helicity more than at the fiber scale.

Our novel comparison framework and simulator-independent metrics also allowed us to directly compare simulated filaments to shapes of actin filaments in cells segmented from cryo-electron tomograms (Serwas et al., 2022) . When processing this dataset, we found that the vast majority of these segmented filaments were uncompressed or of insufficient length. Therefore, we were unable to use this data to evaluate which simulator better captured experimental conditions. These metrics enable quantitative comparison between simulations and additional experimental data of compressed actin filaments as those data become available from electron tomography and microscopy in the future.

Our study reveals some important guidelines for modeling actin filaments and identifies opportunities for further development. In conditions where twist-bend coupling and supertwist behaviors play a significant role, Cytosim may not accurately model filament dynamics without modifications to account for individual monomers or filament normal vectors. On the other hand, ReaDDy is likely able to capture these dynamics, but the significantly higher computational cost may make scaling to larger systems infeasible. Differences in twist-bend coupling between monomer-scale and fiber-scale suggest that lower resolution models, such as network-scale continuum models, may benefit from incorporating terms or adjusting parameters to approximate these individual filament behaviors observed at higher resolutions.

Importantly, these comparisons provide a foundation for automatically inter-converting between both simulators during a multiscale simulation that leverages the benefits of both simulators. *Vivarium* (Agmon et al., 2022) , a software tool for building integrative multiscale models, will enable adaptive switching between Cytosim and ReaDDy. Composing these two models into a hybrid multiscale simulation will theoretically capture monomer-level properties in actin filaments during micrometer-scale cellular processes such as endocytosis with reasonable compute cost and efficiency (Gunaratne et al., 2022) .

Accurately modeling actin filament dynamics is crucial for understanding and predicting cellular structure and function. Comparing different simulation methods reveals their strengths and limitations, while combining them in a modular way could enable us to build on existing knowledge, switch between different modeling methods, and incorporate emerging developments. This multimodal strategy is essential for advancing simulations of complex biological systems and enhancing our understanding of cellular mechanics.

## Methods

Code for the simulation, analysis, and visualization pipeline is available at https://github.com/simularium/subcell-pipeline . The ReaDDy model is available at https://github.com/simularium/readdy-models . The Cytosim model is available at https://github.com/simularium/Cytosim . Simulation data is available on Quilt at https://open.quiltdata.com/b/allencell/tree/aics/subcellular_model_simulations/subcellular_model_simulations_actin_comparison/ .

**Compression simulations** . We simulated the compression of a 500 nm actin filament to 70% of its initial length (350 nm) at four different compression velocities (4.7, 15, 47, and 150 µm/s) with five replicates each. External forces may impose cellular compression rates on the scale of µm/s (Roffay et al., 2021) . We simulated filament compression at rates beyond what is physiologically expected, in order to test the limits of these simulators, within limits of reasonable compute time.

We used *Cytosim* for fiber-scale simulations; representing the filament as a chain anchored by two binding linkers spaced 10 nm apart at each end. One end's linkers were translated linearly for one time step, then allowed to relax for nine, until the filament was compressed. The displacement during each translation step was defined by the compression velocity (2.372 × 10 ^-5^ µm for 4.7 µm/s, 7.5 × 10 ^-5^ µm for 15 µm/s, 2.372 × 10 ^-4^ µm for 47 µm/s, and 7.5 × 10 ^-4^ µm for 150 µm/s), with a time step of 5 × 10 ^-7^ s.

We used *ReaDDy* for monomer-scale simulations; the filament is composed of particles representing an actin monomer, based on actin electron tomography and crystal structure measurements (Fäßler et al., 2020; Oosterheert et al., 2022) using UCSF Chimera (Pettersen et al., 2004) . The actin filament structure was enforced with topology potentials in ReaDDy: harmonic bonds and angles between each actin monomer and each of two neighbors in each filament direction and cosine dihedrals between each set of four consecutive monomers in the filament. Force constants were optimized for stiffness without disrupting structure. We estimated the persistence length for uncompressed ReaDDy simulations after relaxation to be 20.9 ± 14.4 µm (n = 5 filaments × 135 timepoints) compared to the experimentally observed value of 9 - 11 µm for ADP-actin filaments (Bibeau et al., 2023; Isambert et al., 1995; McCullough et al., 2008) . The three monomers at the pointed end were linearly displaced for one time step, then allowed to relax for nine, to match Cytosim's compression method. Monomer-scale simulations used the intrinsic time step of 0.1 ns.

**Simulation infrastructure** . Simulations were run on AWS EC2 m5.large (2 vCPU and 16 GiB RAM) and m5.xlarge (4 vCPU and 32 GiB RAM) instances using Docker images of both models. Approximate wall clock times provided for each compression velocity.

**Table:** 

Compression velocity (µm/s)	Simulated time (ms)	Cytosim wall time (hr)	ReaDDy wall time (hr)
4.7	31.7	15	250
15	10.0	5	80
47	3.17	1.5	25
150	1.00	0.5	8

**Alignment and dimensionality reduction** . Filaments were aligned along the positive y-axis for analysis. For each filament, we calculated the distance of each point in the filament from the origin in the yz-plane, identified the furthest point, computed the angle needed to rotate this point to lie on the positive y-axis, and applied this rotation to all y and z coordinates. Principal Component Analysis (PCA) was performed on these aligned filaments using the Python package *scikit-learn* .

**Visualization** . Simulations were visualized using Simularium (Lyons et al., 2022) , which enabled comprehensive 4D visual assessment of differences in filament behavior between simulators. Interactive visualizations are provided at https://simularium.github.io/subcell-website .

**Declaration of generative AI and AI-assisted technologies** . During the preparation of this work, the authors used ChatGPT-4 to summarize meeting notes, identify author contributions, and reduce word count. After using this tool, the authors verified, reviewed, and edited the contents as needed and take full responsibility for the content of the publication.
